# Emotional intelligence training for pre-service primary school teachers: a mixed methods research

**DOI:** 10.3389/fpsyg.2024.1326082

**Published:** 2024-06-24

**Authors:** Meryem Özdemir Cihan, Mücahit Dilekmen

**Affiliations:** ^1^Department of Primary School Education, Education Faculty, Atatürk University, Erzurum, Türkiye; ^2^Department of Psychological Counselling and Guidance, Education Faculty, Atatürk University, Erzurum, Türkiye

**Keywords:** emotional intelligence, social and emotional learning, emotional intelligence training, emotional intelligence development, pre-service teacher

## Abstract

**Introduction:**

This research devises a training program for developing emotional intelligence focused on social and emotional learning and integrates it into teaching to improve pre-service teachers’ emotional intelligence.

**Methods:**

The study used a embedded mixed design. The quantitative dimension of the study used a controlled quasi-experimental design with pre-test/post-test, and the qualitative dimension used an evaluative case study design. The study was conducted with 73 prospective primary school teachers studying at the faculty of education of a Turkish state university. The study group was formed using purposive random sampling. The sample for the quantitative dimension was composed using probability random sampling, whereas the sample for the qualitative dimension was composed using purposive sampling. The program was administered to the experimental group for 10 weeks. Quantitative data were obtained using the Bar-On EQ-i, and qualitative data were obtained using a semi-structured interview questionnaire and participant diaries. Quantitative data were analyzed using normality analysis and one-factor analysis of covariance, whereas qualitative data were analyzed using descriptive and content analysis.

**Results:**

The results found the developed training program to be an effective approach to improve emotional intelligence among pre-service teachers. Moreover, data obtained from documentary sources and focus group interviews during and after the application of the program confirmed and adequately explained the quantitative results.

**Discussion:**

In line with the purpose of the study, the findings obtained from the quantitative part of the study showed that the implementation of the training program for the development of emotional intelligence focused on SEL was an effective approach in increasing the emotional intelligence levels of the pre-service teachers in the experimental group. Similar research results also support that pre-service teachers’ emotional intelligence can be improved with additional intervention practices without affecting their curriculum.

## 1 Introduction

Emotional intelligence is a cross-section of interrelated emotional and social competencies, skills, and facilitators that determine how effectively we understand and express ourselves and cope with daily demands (Bar-On, 2006). Although there are many definitions of what emotional intelligence is, the most comprehensive and widely accepted definition was put forward by Mayer and Salovey (1997), according to whom emotional intelligence is the ability to monitor one’s own and others’ feelings and emotions, to discriminate between them, and to use this information in one’s thoughts and actions.

When the literature is examined, it is seen that emotional intelligence is addressed with various variables. Most of the research results provide evidence for the positive effects of emotional intelligence in many areas. For example, there are many studies on the role of emotional intelligence competence in achieving academic success (Gilar-Corbí et al., 2018; Howard and Cogswell, 2018; Petrides et al., 2018). There are also studies showing that there is a relationship between academic achievement and emotional intelligence competence (Nasir and Masrur, 2010; Grehan et al., 2011; Petrides et al., 2018; Wolf et al., 2018; Jan et al., 2020; Pozo-Rico and Sandoval, 2020).

There are numerous studies on the benefits of teachers’ emotional intelligence in the classroom (Extremera and Fernández-Berrocal, 2004; Brackett et al., 2010; Schoeps et al., 2019). Research has shown that teachers with higher levels of emotional intelligence use more constructive strategies for conflict management (Valente and Lourenço, 2020). It is thought that teachers’ developed emotional intelligence may help them cope with burnout (Chan, 2006). Based on these findings, emotional skills programs should be included in their academic education in order to improve the emotional intelligence of teachers/pre-service teachers and provide them with conflict resolution strategies (Valente and Lourenço, 2020). However, implementation programs should be well analyzed in order to train teachers who will implement emotional intelligence training in the classroom (Pozo-Rico and Sandoval, 2020).

Although emotional intelligence skills are needed at every stage of life, it is crucial to develop these skills during primary school education, a critical period for personality and skill development. This primarily requires sufficiently developed emotional intelligence skills among primary school teachers, who closely engage with children in such critical developmental periods. Hence, it is necessary to develop emotional intelligence among pre-service teachers with a focus on SEL. Indeed, emotional intelligence predicts career success and life satisfaction (Amdurer et al., 2014) and allows developing various competencies, including better problem solving and coping with problems and combating anxiety (Bastian et al., 2005; Bar-On, 2006).

Emotional intelligence training can be provided at any educational setting and can be adapted to any age group. This training helps build common sense and reduce social problems. Many researchers have developed programs/models for emotional intelligence development and have shown that emotional intelligence can be developed at any age, leading to positive results in many aspects (Mayer and Salovey, 1997; Goleman, 1998; Shapiro et al., 1998; Bar-On and Parker, 2000; Mayer et al., 2001). Emotional intelligence training also helps individuals establish socially and emotionally healthy relationships and positively impacts individuals’ quality of life of (Mayer and Salovey, 1997; Shapiro et al., 1998). Research findings support the idea that emotional intelligence can be strengthened through education (Brackett and Katulak, 2006; Clarke, 2010; Hen and Goroshit, 2010).

Curricula from primary school to university seem to overemphasize cognitive skills to the detriment of emotional intelligence skills. As scholars have long observed, emotion is one of the most disregarded dimensions in research and practice for educational change (Hargreaves, 1998a,b; Spillane et al., 2002; Zembylas, 2002). Recognition of the role of emotions in teaching and learning in higher education is long overdue (Mortiboys, 2013). Focusing only on academic results leads to neglect of important areas such as social–emotional and behavioral aspects (Day et al., 2007). Studies on the development of personal emotional intelligence through training programs targeting the training needs of teachers in educational settings are still scarce (Haskett, 2003; Drew, 2006; Jennings and Greenberg, 2009). Zembylas (2005) examined the relations between pedagogical content knowledge and emotional knowledge in teaching and learning and mentioned that complementary research focusing on the cognitive aspects of teacher knowledge is needed to analyze the emotional dimension of teachers in teaching and learning.

## 2 Theoretical background and research questions

### 2.1 Social and emotional learning

The SEL refers to the integration of thoughts, emotions, and behaviors (Brackett and Rivers, 2014). In other words, it is the process through which children and adults acquire and effectively apply knowledge, skills and attitudes needed for supporting healthy identity formation, understanding and managing emotions, setting and achieving positive goals, empathizing, and developing positive relationships (Kress and Elias, 2006). SEL is defined as an integral part of education and human development. At its core are five interrelated social and emotional competencies (self-awareness, self-management, social awareness, relationship skills, and responsible decision-making) that support learning and development (Collaborative for Academic, Social, and Emotional Learning, 1994).

Teachers play a major role in the acquisition of these skills in the school environment. Socially and emotionally competent teachers create a positive environment in the classroom. Thus, the teacher who responds to students’ emotions by using appropriate verbal and physical expressions will be able to prevent unwanted behaviors and manage the lesson effectively. A teacher who displays a warm and compassionate approach that supports students’ positive emotions can increase students’ learning capacity, whereas a teacher who despises and judges students’ behavior can irreversibly destroy the bond with the student (Brackett et al., 2010). Goleman (1997) stated that learning will not occur in a student who develops negative emotions. Thus, teachers as well as students are expected to be equipped with fully developed social and emotional skills.

### 2.2 Social and emotional learning programs

As schools have come to recognize the social and emotional needs of students, they have discovered that these skills can be taught and learned alongside traditional content knowledge. Increasing interest in this field has prompted a number of questions including “What is SEL? Is it important? How does it happen? How can it be integrated into school curricula?” (Elbertson et al., 2010). Over time, numerous programs have been designed in this field (e.g., CASEL, PATHS, RC, The 4Rs Program, RULER, and The Six Seconds EQ Model). Some programs have solid foundations that include all components of SEL, while others have tried to address them individually.

Most countries have made great efforts to integrate SEL competencies into school curricula (e.g., U.S. Department of Education, 2016). There is an understanding that some students are socially and emotionally deficient, which reduces their sense of belonging to school (Corcoran et al., 2018). Although there has been pressure for schools to focus more on academic achievement, the adoption of the Every Student Succeeds Act (ESSA, 2015) in the United States has raised awareness of the need for fostering social and emotional competencies (Corcoran et al., 2018). Some studies have examined the effects of SEL programs on various variables. For example, many programs have been developed to reduce and prevent violence, reduce bullying and victimization, reduce problematic behaviors and delinquency, and raise awareness to increase student achievement. Corcoran et al. (2018) conducted a meta-analysis of 40 studies on SEL practices and examined the overall outcomes of these school-based programs and their impacts on reading, mathematics, and science in pre-K-12 grades. They found that these practices had positive effects on reading and mathematics and a small effect on science when compared to traditional methods. In conclusion, these programs have been found to yield much better results than traditional programs (Durlak et al., 2011), result in academic achievement at least twice as high as in other programs (Dix et al., 2012), and lead to a decrease in drug use and delinquency (Battistich et al., 2000).

Primary schools in Türkiye have quite heterogeneous classrooms due to various factors, including age differences, individual and socio-cultural backgrounds, and presence of refugee children (Kamışlı et al., 2020). Within such structures, children need teachers who can recognize their talents and potential and respond to their needs (Nieto, 2003). In Türkiye, objectives for social and emotional skills for students in formal education are embedded in the learning outcomes of existing curricula, which aims to develop cognitive, social, and emotional skills simultaneously (Türnüklü, 2004). The first program designed to develop Turkish students’ social–emotional skills was the “Emotional and Social Development Course” program, which became effective in 2012 after being adopted by the Board of Education and pioneered studies on SEL in Türkiye. However, this program is mainly intended for developing social–emotional skills among gifted students. Efforts for developing social–emotional skills among other students are mainly concentrated in the field of psychological counseling and guidance (Suna et al., 2021).

Looking at the Turkish context, four-year undergraduate programs of teacher training institutions are constantly changing and being updated (as in 1997, 2006, 2009, and 2018). Until 2018, cognitive domain courses were predominant in the curricula. In the 2018 curriculum, courses were divided into three groups: pedagogical knowledge, general culture, and content knowledge, with the bulk of the curriculum composed of content knowledge courses (Kılıç Özmen, 2019). In a study conducted by Çelik (2020), curricula were evaluated by graduates who highlighted the insufficiency of psychological and social development courses and lack of training in classroom management and guidance.

It is crucial to teach children SEL skills during formative periods. Many studies have shown that delayed interventions fail to create the desired impact on child development (Bredekamp and Joseph, 2011; Bergen and Robertson, 2012; Murano et al., 2020). Thus, primary school teachers are expected to become competent in recognizing children and assessing their development in a timely manner (Kamışlı et al., 2020). Leadership skills such as being open to change, continuous professional development, empathy, communication, problem solving, and recognition of social skills through exemplary character and expertise should be part of the requirements of the teaching profession (Susar Kırmızı and Yurdakal, 2020). Efforts made by academia and various organizations in the field of emotional intelligence have shown that emotional intelligence can be developed in adults too (Cherniss and Goleman, 2001; Slaski and Cartwright, 2003; Bar-On, 2006; Boyatzis, 2009). Based on these results and the links between emotional intelligence and effective teaching, some experts have suggested that teachers can benefit from developing their emotional intelligence skills (Haskett, 2003; Brackett et al., 2009). All these reports support the idea of teacher-targeted emotional intelligence development as an important element of SEL programs (Weare and Gray, 2003; Brackett et al., 2007, 2009; McCown et al., 2007; Palomera et al., 2008).

### 2.3 Current study objectives and research questions

When the studies of the last 25 years are examined, while emotional intelligence education is at the forefront in a wide range from kindergarten to high school, it has been stated that there is a deficiency in the design and implementation of emotional intelligence education by academicians in undergraduate and graduate education (Zeidner et al., 2002; Abraham, 2005; Holt and Jones, 2005; Beard et al., 2008; Pool and Qualter, 2012; Bonesso et al., 2013; Schutte et al., 2013; Mattingly and Kraiger, 2019).

Emotional intelligence training is very useful and important for educators as well as students. Students can be more active and successful academically and socially with their emotional intelligence skills and can establish strong social relationships. In this way, teachers can conduct more effective and enthusiastic lessons by reducing unwanted behaviors in the classroom (Stone-McCown et al., 1998). There is also evidence that emotional intelligence training can reduce cortisol levels and stress and thus increase life satisfaction (Nelis et al., 2011). Researchers working on learning and emotions have argued that teaching is one of the most stressful professions and that it may be possible to cope with a stressful environment through training in emotional abilities (Palomera et al., 2008). Farber (1991) emphasized the need to understand and address teacher stress and burnout in order for American education to be successful. He also suggested that the development of individual strategies to enable teachers to manage stress is a prerequisite for success in education. It is emphasized that emotional intelligence can help one manage emotions associated with negative events and understand the emotions of others (Sergi et al., 2021). It is noteworthy that teachers/pre-service teachers with high emotional intelligence are more likely to have positive emotions and their life satisfaction increases in parallel (Deng et al., 2023).

Learning and teaching are not only about knowledge, cognition and skills but also emotional practices (Claxton, 1999; Hargreaves, 2001). The social aspects of the learning environment also significantly increase the learning rate. If you are responsible for helping others learn, you need to be able to recognize and work with this emotional component of the teaching-learning exchange. In short, teachers need to use emotional intelligence. It has been emphasized that cognitive, social and emotional skills have an important role in teacher training (Cefai and Cooper, 2009). It has been suggested that there is a relationship between the quality of teachers’ work and emotional intelligence (Sutton and Wheatley, 2003; McCown et al., 2007). The level of bonding, communication and respect between a child and a teacher affects the child’s brain development (Kusche and Greenberg, 2006). Students who have warm, supportive, positive and respectful interactions with their teachers have higher academic motivation and engagement (Ryan and Patrick, 2001). Similarly, in environments where positive relationships develop, students experience a sense of belonging to the school and community and exhibit better academic performance (Osterman, 2000).

There is a broad consensus on the importance of developing emotional intelligence (Elias and Clabby, 1992; Greenberg et al., 1995; Bar-On and Parker, 2000; Nelson and Low, 2003; Domitrovich et al., 2007; Palomera et al., 2008; Nelis et al., 2009). Of course, achieving this development requires an effort for implementation at all levels from primary school to university. Although it is possible to show this effort, organizations may face great difficulties (Zins et al., 2004). Studies have shown that when businesses want to employ students in fields that require emotional intelligence skills, students do not have sufficient skills in controlling their emotions, working in teams, managing other people and adapting to changes (Pertegal-Felices et al., 2011, 2014). From this point of view, it seems necessary to integrate social–emotional skills training into the curriculum by starting social–emotional skills training from the beginning of teaching in order to ensure the effective professional development of teachers (Palomera et al., 2008; Pertegal-Felices et al., 2011).

When the relevant literature is examined, studies on the development of emotional intelligence have been carried out mostly in the following areas:

- *Different groups;* mothers, diabetic patients, general practitioners, hemodialysis patients (e.g., Karahan and Özçelik, 2006; Boylan and Laughrey, 2007; Balcı, 2016; Shahnavazi et al., 2018).
- *University students’ group;* agriculture, dentistry, science and literature, education, engineering, nursing, social services, medicine, psychology, business administration (e.g., Potter, 2005; Chang, 2006; Fletcher et al., 2009; Nelis et al., 2009; Jdaitawi, 2012; Erkayıran, 2016; Orak et al., 2016; Pertegal-Felices et al., 2017; Geßler et al., 2020).
- *K-12 student group;* age and education groups such as kindergarten, primary school, middle school, high school, special education, adolescents, etc. (e.g., DiNatale, 2000; Gerber, 2004; Brackett et al., 2012; Arslan and Güven, 2015; Şahin and Ömeroğlu, 2015; Zorlu Uğur, 2015; Gürsoy, 2016; Viguer et al., 2017; Altunbaş, 2018; Cerit, 2018; Mavruk Özbiçer, 2018; Coşkun and Öksüz, 2019).
- *Teacher groups;* (e.g., Hen and Sharabi-Nov, 2014; Dolev and Leshem, 2016; Dolev and Leshem, 2017; Turi et al., 2017; Sarısoy and Erişen, 2018; Martyniak and Pellitteri, 2020; Valente and Lourenço, 2020).
- *Pre-service teacher groups;* (e.g., Joshith, 2012; Gilar-Corbí et al., 2018).

The majority of these studies were conducted experimentally. Qualitative or mixed design studies are quite few. It has been observed that the experimental studies were mostly conducted by developing and implementing a program for emotional intelligence and examining its effectiveness with pre-test/post-test analysis. For example, Clarke et al. (2015) found that 49 of the 94 studies they examined did not have a comparison group. Therefore, it is unclear whether positive outcomes can be attributed to the intervention. On the other hand, it should be underlined that in most of these studies, an improvement was achieved with the emotional intelligence trainings developed and implemented. It is seen that these results have also been revealed through meta-analysis/systematic review (Emerson et al., 2017; Hwang et al., 2017; Oliveira et al., 2021) and scoping review (Turner and Stough, 2020) studies. However, these studies are also quite limited.

Upon examining the results of the SEL intervention program, it is noteworthy that Corcoran et al. (2018) found that actual experiments produced smaller effect sizes than quasi-experimental studies. However, while there are many quantitative studies focusing on the outcomes of SEL interventions, qualitative research can provide a better understanding of the black box of these programs (Corcoran et al., 2018). More reliable and valid results can be obtained by conducting both quantitative and qualitative applications together and synthesizing their results. While studies that blend quantitative and qualitative findings on such a topic are extremely significant, their scarcity is an important problem.

As a result, social–emotional learning programs are increasingly being implemented and have various positive effects on students’ academic and social behaviors (Freedman and Jensen, 2008; Durlak et al., 2010). However, these programs usually constitute only a small part of the school curriculum. Moreover, even in countries that care about students’ emotional intelligence development, there has been less focus on teachers’ own development (Jennings and Greenberg, 2009).

Faculties of education tend to focus on actual behavior/actions rather than the emotions behind the actions of pre-service teachers in order to meet the standards of professional teacher preparation, development and assessment. To date, in the Turkish context, there is no formal emotional intelligence education in the curriculum for education faculty students. Teacher training programs mostly focus on ways to increase pedagogical skills and content knowledge for pre-service teachers with little emphasis on improving their emotional and social skills. However, pre-service teachers should be able to improve whatever social–emotional deficits they have before they enter the classroom. Although the findings in the literature show that university students’ emotional intelligence development is provided through emotional intelligence training programs, emotional intelligence interventions applied to pre-service teachers in real-world settings are very few. Therefore, it shows that it is very important to develop emotional intelligence by integrating it with the educational programs. At the same time, it reinforces the idea that pre-service teachers can be equipped with the emotional competencies expected to have in the real classroom environment. In addition, it has a critical importance in terms of providing information about how to develop the program for pre-service teachers and how to measure its effectiveness. While studies that blend quantitative and qualitative findings on such a topic are extremely important, their scarcity is another problem (Corcoran et al., 2018). Therefore, the purpose of this study is to examine whether designing a social–emotional learning-oriented training program for emotional intelligence development and integrating it into teaching is effective in developing pre-service teachers’ emotional intelligence. In addition, it is also aimed to provide guidance on how such a program can be integrated and implemented in the courses. Consistently, the study will answer the following research questions:

1. What is the impact of the emotional intelligence training on pre-service teachers’ emotional intelligence levels?
2. How do pre-service teachers evaluate the emotional intelligence training throughout the process?
3. How do pre-service teachers evaluate the emotional intelligence training after completing the process?
4. What conclusions can be drawn from the integration of quantitative and qualitative findings?

The results of this study are important as they provide suggestions that policymakers, practitioners, and other researchers can use in their practices in schools.

## 3 Methods

### 3.1 Participants

The sample of the study comprised the students of a four-year undergraduate program of primary teaching at the Faculty of Education, Atatürk University, a state university in Türkiye. To ensure teachers’ effective professional development, it is necessary to integrate social–emotional skills training into the curriculum from the beginning of teacher education (Palomera et al., 2008; Pertegal-Felices et al., 2011). This primarily requires sufficiently developed emotional intelligence skills among pre-service primary school teachers, who closely engage with children in such critical developmental periods. These factors were instrumental in the determination of the sample.

**Figure 1:**
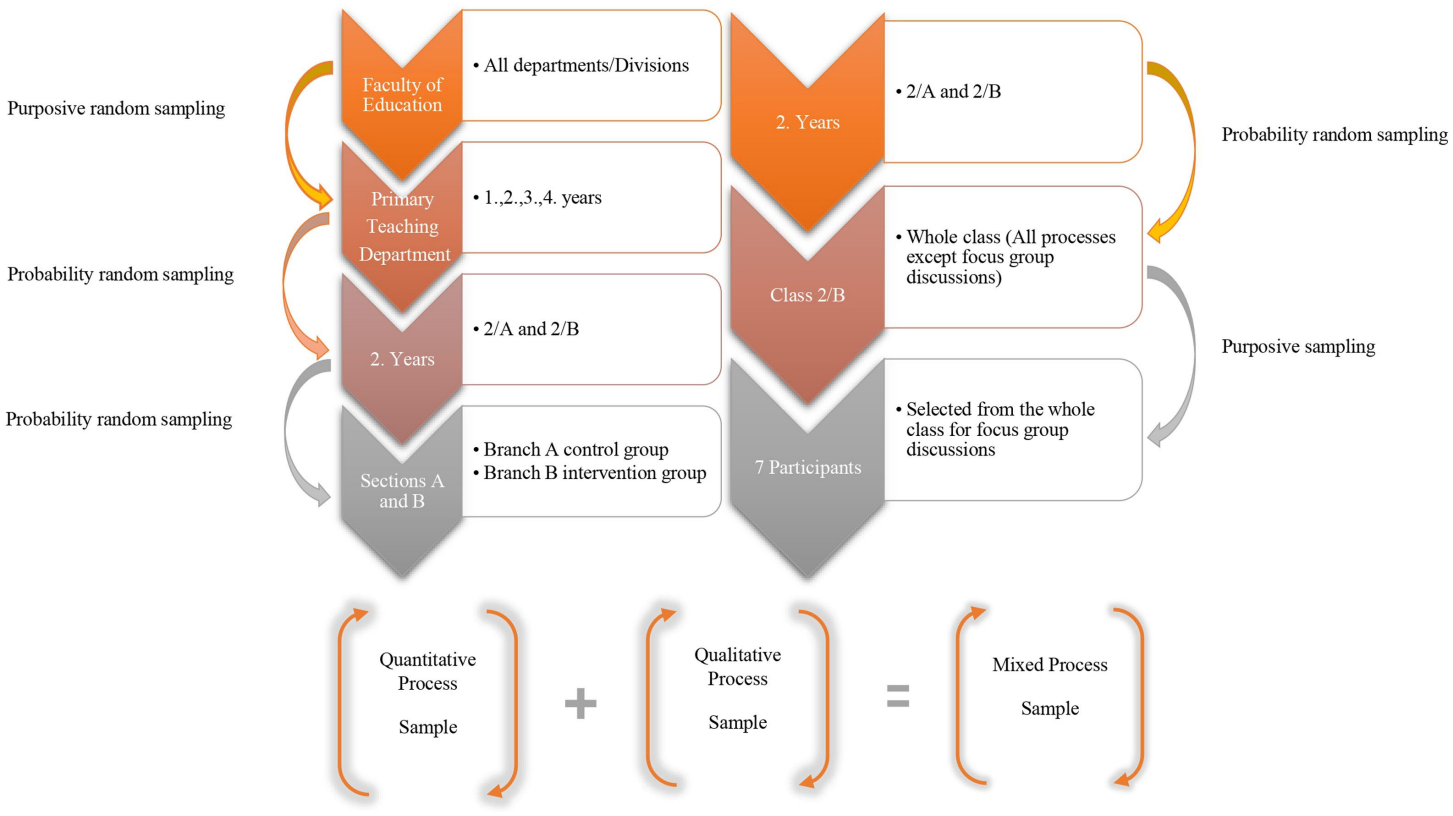
Participant selection processes from the population to the sample.

The study used both probability and purposive sampling techniques. Probability sampling strategy was used for the quantitative process and purposive sampling for the qualitative process. Additionally, the intervention group sample was determined using identical and nested sampling procedures. The criterion for selecting participants for purposive sampling was full attendance and active participation in all sessions. As it is best to select the sample from the same population (Creswell, 2014), both samples were selected from the same population. The purpose of using probability-based random sampling is to generalize from the general population to the sample and to control for selection bias (Marshall, 1996). Purposive random sampling provides more extensive data than those yielded by random sampling and improves the credibility of the research (Teddlie and Tashakkori, 2009; Flick, 2014). Participation in the study was voluntary, and participants were asked to read and approve the informed consent form. The study received a general comprehensive ethics committee approval from Atatürk University Rectorate, Social and Human Sciences Ethics Committee, and Educational Sciences Unit Ethics Committee to cover the use of scales, recording observations and interviews, and use of the products generated during program (Decision No: 04). Participants were informed of their rights and obligations relating to the process. A flowchart showing the sampling steps is presented in Figure 1.

According to Figure 1, during the quantitative process, the department of primary teaching was selected from the population of the faculty of education through purposive random sampling. Then, of the four grades in the department, the second-year grade was selected using probability random sampling. The second-year grade has two sections, A and B. Of these sections, Section B was selected as the experimental group and Section A as the control group using random allocation lottery. However, in quasi-experimental studies, which are used in educational research due to the impossibility of unbiased allocation of individuals to groups in school and classroom settings, unbiased allocation to existing classrooms is not possible. Two groups with similar qualifications and sufficient number of participants were included to ensure internal validity during the quantitative process. Both groups have similar qualitative characteristics including having similar GPS, similar scores in university admission, and the distribution of female and male participants (leading to identical groups). The experimental group comprised 32 participants (8 males, 24 females); the control group comprised 41 participants (7 males, 34 females). Participants in both the groups were aged between 18 and 24 years. For the qualitative process sampling, the nested sampling procedure was used whereby seven participants were selected for focus group interviews using purposive sampling from among the participants in the experimental group (Branch B) which had been assigned by probability random sampling.

The study results suggested that the sample size might be sufficient when using the G*Power 3.1.9.7 program. The sample calculation for ANCOVA was performed based on a research design with two groups (experimental and control) and two measurements (pre-test and post-test). Using an effect size of 0.595 (*f* = 0.595), a 5% margin of error (*α* = 0.05), and 95% power (1−*β* = 0.95), the total sample size was determined to be 39 (approximately 19–20 participants per group).

### 3.2 Measures

#### 3.2.1 Data collection tools for the quantitative dimension

The Bar-On Emotional Quotient Inventory, used in the quantitative dimension, has been used in many previous studies in Türkiye and has been found sufficient, valid, and appropriate in the context of measuring emotional intelligence levels. The inventory consists of five main dimensions with sub-dimensions (personal skills, interpersonal skills, compatibility, coping with stress, general mood. See Table 1 for the sub-dimensions). It was adapted into Turkish by Tekin Acar (2001) and reduced from 133 items to 87 items. Mumcuoğlu (2002) studied the linguistic equivalence, reliability, and validity of the Turkish version. Item 80, which Tekin Acar excluded from the analysis due to the low reliability coefficient of the statement “I know that it is difficult for me to control my anxiety” in the stress management dimension, and item 88, which includes the statement “I answered the above statements sincerely and truthfully,” which he added at the end of the inventory were excluded from the analysis. Finally, 86 items of the inventory were used and analyzed for reliability. Cronbach alpha values were calculated for both the total inventory and the sub-dimensions separately with pre-test and post-test. The values were as follows: *α* = 0.88 for the sum of the dimensions at pre-test and *α* = 0.87 for the sum of the dimensions at post-test; *α* = 0.80 for the intrapersonal skills dimension at pre-test and *α* = 0.80 for the intrapersonal skills dimension at post-test; *α* = 0.82 for the interpersonal skills dimension at pre-test and *α* = 0.80 for the interpersonal skills dimension at post-test; *α* = 0.67 for the adaptability dimension at pre-test and *α* = 0.66 for the adaptability dimension at post-test; *α* = 0.68 for the stress management dimension at pre-test and *α* = 0.69 for the stress management dimension at post-test; and *α* = 0.80 for the general mood dimension at pre-test and *α* = 0.79 for the general mood dimension at post-test. As these values were similar to the results of previous validity and reliability studies and were within the limit values, the scale was used without any change. Furthermore, to ensure the reliability of the results and to accurately determine the discrimination of the development in the process, the same scale was used for the pre-test and post-test, without any addition or deletion of items during the process. In the study’s inferential statistics, analysis was conducted using the total emotional intelligence scores.

**Table 1:** Bar-On’s emotional intelligence model.

Main dimensions	Sub-dimensions
Personal skills	Independence
	Self-actualization
	Self-confidence
	Self-respect
	Emotional self-awareness
Interpersonal skills	Social responsibility
	Interpersonal relationships
	Empathy
Compatibility	Flexibility
	Measure of reality
	Problem solving
Coping with stress	Stress tolerance
	Impulse control
General mood	Happiness
	Optimism

#### 3.2.2 Data collection tools for the qualitative dimension

##### 3.2.2.1 Semi-structured interview questionnaire

Semi-structured interview questionnaires were developed by the researcher and finalized after receiving expert opinion. One of the questionnaires was designed for use at the end of each session during program and another was designed for use at the end of the program for focus group interviews with the participants selected from the experimental group. Each questionnaire used during the program comprised questions related to the session titles and contents. The questionnaire used at the end of the program consists of questions that inquire about knowledge, skills, and positive and negative experiences at the end of the process. The participants of the focus group interviews conducted during and after the intervention were the same subjects and the examples quoted from the interviews were coded and used in the results section as follows: EP1, EP2… EP7 (Experimental Participant = EP).

##### 3.2.2.2 Participant diaries

At the beginning of the intervention, the researcher distributed diaries to the participants in the intervention group for them to evaluate each session. These diaries were used at the end of each day of the program by participants to describe the sessions in detail from beginning to the end, to indicate which interventions contributed to their development positively or negatively and to include their opinions and suggestions. A total of 32 participant diaries were used in the study. The diaries had 80 facing pages in total. The average number of pages used by all participants was 62. The results section provide some visuals from the participant diaries, coded as PD1, PD2… PD32 (Participant Diary = PD).

### 3.3 Procedure and design

#### 3.3.1 Research design

**Figure 2:**
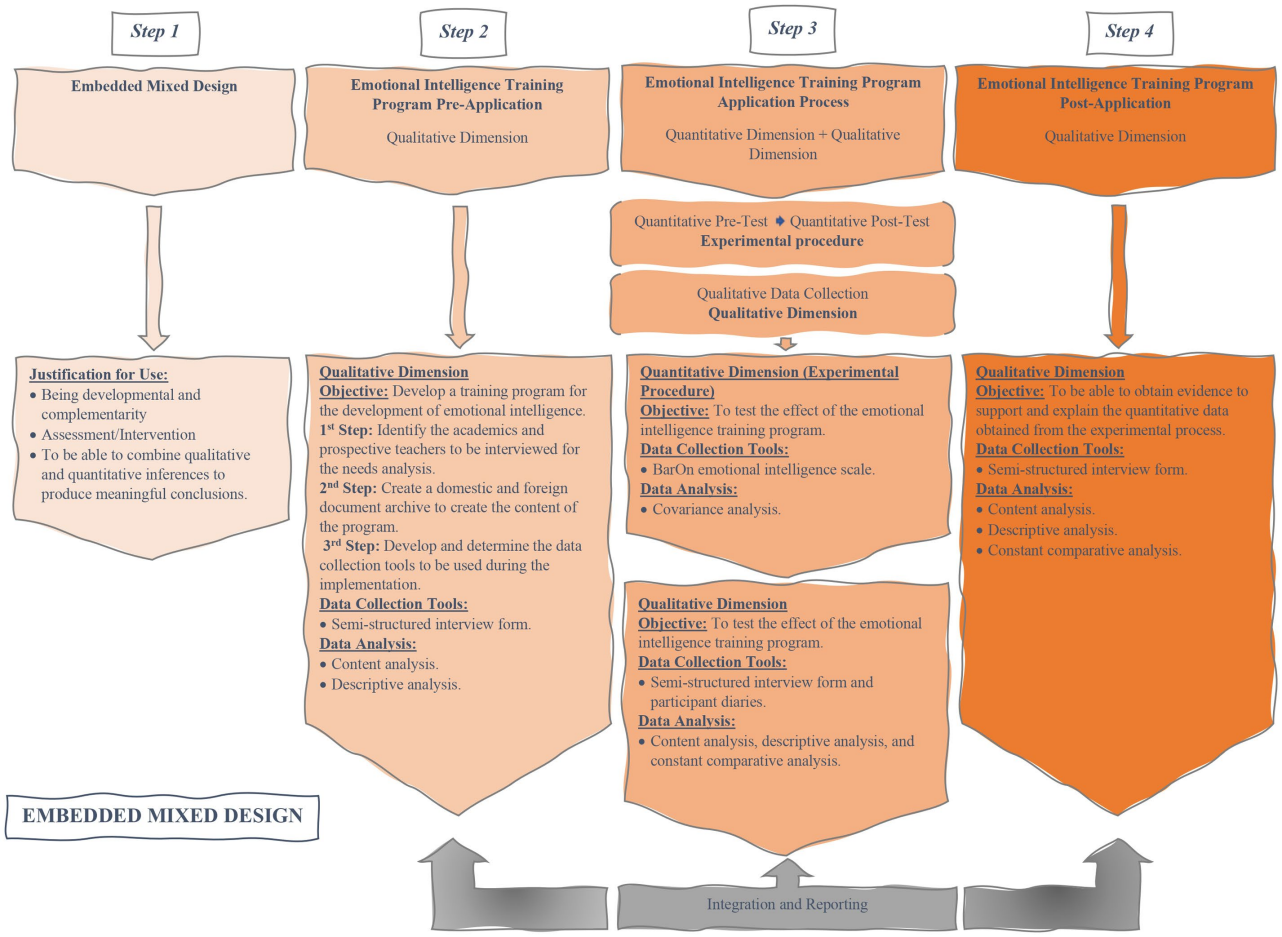
Embedded mixed design process flowchart.

This study used a embedded mixed methods design, which allows the use of different methods and analyses. The quantitative dimension used a controlled quasi-experimental design with pre-test and post-test, whereas the qualitative dimension aimed to perform an in-depth analysis of the training program in a real setting and, to this end, used the “evaluative case study” design, a type of case studies defined by Merriam (2013). The process flow chart showing the steps of mixed methods research is presented in Figure 2.

Table 2 presents the implementation process and procedural steps for both the experimental and control groups.

**Table 2:** Implementation process and procedural steps for experimental and control groups.

Control group implementation process	Experimental group implementation process
The course “Instructional technologies and material design” (weekly 4 h/3 credits = 14 weeks), a 2nd year course of primary teaching in the spring semester of academic year 2018–19, was selected for the implementation of the program.	
Since the structure of the course is predominantly practice-oriented, the theoretical foundations of the course were delivered to both groups for 1 month with the same content.	
Both the experimental and control groups received 16 h (960 min) of theoretical training on the course content.	
The control group received the existing training program, while the experimental group received a 40-h (2,400 min) training program focused on developing emotional intelligence through social and emotional learning.	
During the first 4 weeks of the semester, all the theoretical parts covering the subject, objectives, and achievements of the course were covered. During these 4 weeks, the knowledge levels of the pre-service teachers were measured using quizzes and question and answer techniques at the end of each lecture, and areas for improvement were reviewed to solidify the knowledge.	
In the control group, as implemented within the current education program, the individuals who were determined by lot for each week were assigned to the subjects within the units within the scope of the Turkish, Social Sciences, Science, Mathematics and Life Sciences courses in the curriculum developed by the Ministry of National Education for primary school 1st, 2nd, 3rd, and 4th grades (For example; Science: States of Matter, Turkish: Synonyms and Antonyms, Mathematics: Angles, Social Studies: Production/Consumption, Life Science: Transportation Vehicles) were determined by the number of people and distributed to the pre-service teachers by lot. Until the end of the semester, each pre-service teacher prepared a lesson plan for the grade level of the subject they had taken made a short lecture addressing the same level and made a presentation using the materials they had developed for that subject.	During the first week of the 10-week process, the “sharing circle” was introduced and piloted, and all the rules for the process were explained to the students in detail.
	The implementation process started with the program introduction and was completed in 10 weeks.
	The eight emotional intelligence skill areas were distributed over the remaining nine weeks (communication skills were divided into two weeks due to the extensive scope of the subject).
	During the process, a focus group interview was conducted at the end of each session using a semi-structured interview form.
	At the end of the implementation process, a focus group interview was conducted with a semi-structured interview form consisting of questions that helped to reveal the knowledge, skills and positive and negative experiences obtained.
Materials were designed according to the topics.	Materials were designed according to the session topics.
Both the experimental and control groups were administered the Bar-On emotional intelligence scale as a pre-test at the beginning of the course (One semester = 14 weeks) and as a post-test at the end.	

In addition, the program implemented in the experimental group was integrated into the curriculum in accordance with the objectives and outcomes of the course without interfering with the academic performance of the pre-service teachers and without overloading the out-of-class studies. The theoretical and practical parts of the course were organized in a purposeful way in both groups. Appropriate materials were designed for the topics. All of the theoretical part of the course was covered for the subject, objectives and achievements of the course. At the end of each lecture in these 4 weeks, the knowledge levels of the pre-service teachers were measured with quizzes and question and answer techniques, and the missing parts were reviewed and the information was tried to be made permanent. As a result of the exams, the academic success levels of both groups are close to each other (Average score of the control group out of 100: 88.07, Average score of the experimental group out of 100: 88.75). While the program applied in the experimental group was integrated into the curriculum, it was tried not to allow any deformation in the purpose and content of the course. In the experimental group, the ‘application program’ and the ‘instructional technologies and material design’ course were made compatible with each other. In the experimental group, attention was paid to ensure that the environment in which the practices would be carried out had the characteristics that would be suitable for circle activities (no fixed desks, a large, spacious, brightly lit area large enough for group activities, audio and video equipment) and the most suitable place was selected.

#### 3.3.2 Process of development and implementation of SEL-oriented emotional intelligence development training program

There are many arguments that form the basis for designing a training program for the development of emotional intelligence. These arguments range from theories put forward by emotional intelligence experts to programs developed and implemented worldwide, from emotional intelligence inventories to activities that provide social–emotional development. The program was developed by considering the emotional intelligence development levels of both children in primary education and primary teachers. Additionally, needs assessment interviews were conducted with five academics from different branches with previous research/studies on emotional intelligence and interest in this concept to seek expert opinion and suggestions for designing the intervention process and to identify the gaps in the field. Moreover, needs assessment interviews were conducted with 10 pre-service teachers studying primary teaching for the same purpose. The contents of the sessions were prepared based on the characteristics of the integrated course and material design dimensions. Many materials were designed for different components of social–emotional skills as part of the activities. The primary aim of the program is to reveal the existence of practices that can help individuals develop emotional intelligence skills, be it children of primary school age or individuals studying at university and to make these practices an integral part of curricula. To this end, the researcher accessed most of the national and international literature on the development of emotional intelligence (e.g., Mayer et al., 2000; Cherniss and Goleman, 2001; Boyatzis et al., 2002; Bar-On, 2006; Brackett et al., 2007, 2012; Boyatzis, 2009; McEnrue et al., 2009; Clarke, 2010; Durlak et al., 2011; Zorlu Uğur, 2015; Dolev and Leshem, 2016, 2017; Machera and Machera, 2017; Pertegal-Felices et al., 2017; Turi et al., 2017; Viguer et al., 2017; Cerit, 2018; Gilar-Corbí et al., 2018; Mavruk Özbiçer, 2018; Sarısoy and Erişen, 2018; Coşkun and Öksüz, 2019). Models and programs developed for emotional intelligence skills (e.g., CASEL, PATHS, RC, the 4Rs Program, RULER, and The Six Seconds EQ Model) were reviewed. Activities found in the reviewed studies mostly comprised role plays, group discussions, readings, video clips, case studies, group tasks, reflective writing diary, demonstration and role play, games, sensitivity training, interactions, peer feedback, and diaries. Similar activities were designed for the intervention program in this study.

The study was based on Bar-On’s model of emotional intelligence, which places social–emotional intelligence at the forefront. Bar-On (2006) found that individuals with high emotional intelligence are more resilient to life challenges and better equipped to cope with stress and problem-solving. Conversely, those with low emotional intelligence experience more emotional problems and are less successful in coping with stress and problem-solving. Bar-On used his own developed Bar-On Emotional Intelligence Scale (Bar-On EQ-i) to test his model. The Bar-On EQ-i is a reliable scale for measuring emotional intelligence, with good internal consistency (Tommasi et al., 2023). Table 1 presents a visualization of information about Bar-On’s emotional intelligence model.

The developed program is based on “humanistic design,” which is a student-centered curriculum design approach. Humanistic design offers a program structure organized by activity and addresses the intrinsic characteristics of the individual. The aim of this design is for individuals to recognize and develop themselves. This involves various methods and techniques such as learning activities, problem solving, discussion, brainstorming, creativity, educational games, drama, discovery, and invention. At the end of this in-depth review and execution of the whole process in grounding the intervention program, the concepts and social and emotional skills were restructured and gathered in a common pool.

Information on the eight skill areas identified for emotional intelligence and their implementation objectives are presented in Table 3.

**Table 3:** Emotional intelligence skill areas and implementation objectives for sessions.

Sessions	Objectives
Self-awareness and self-consciousness	Being aware of individual differences. Being aware of the thoughts and feelings of others. Being aware of strengths and weaknesses.
Recognizing and managing emotions	Creating a vocabulary for emotions. Identifying positive and negative emotions. Understanding the background of emotions. Identifying experiences that make you feel happy and experiences that make you feel sad. Learning a constructive way to deal with anger. Knowing that it is normal to feel every emotion. Learning how to be emotionally strong.
Communication skills	Learning active listening and speaking. Recognizing healthy and unhealthy ways to communicate. Gaining the ability to make sense of verbal and non-verbal messages. Being aware of communication barriers. Understanding the importance of face-to-face communication. Recognizing the relationship between listening and empathic skills.
Empathy skills	Recognizing non-verbal behavior. Being able to feel other people’s perspectives and emotions on events. Being able to communicate empathetically. Knowing that others can have their own feelings, too. Understanding the relationship between empathy and active listening.
Decision-making skills	Learning decision making and problem-solving steps. Being able to recognize critical decision-making processes. Being able to recognize how decisions affect oneself and others. Recognizing how personal beliefs and desires influence the decision-making process. Considering the consequences of difficult decisions.
Problem/conflict-solving skills	Understanding the nature of conflicts. Seeing the types of conflicts. Learning basic strategies that can be used in problem/conflict solving. Being able to see the possible consequences of the approaches taken in the conflict resolution process.
Stress coping skills	Learning about stress and situations that cause stress. Learning ways to cope with stress. Being able to control thoughts and behaviors caused by stress.
Group work and cooperation	Learning the benefits of working with others. Learning ways to make decisions together with the group. Being able to choose tasks to do with other group members. Learning the importance of achieving common purpose by collaborating with others.

Some of the activities and practices found in the literature were revised and some were designed from scratch by the researcher to cover the eight skill areas for SEL and emotional intelligence (based on the Bar-On model of emotional intelligence). The activities were also reviewed and revised by two professors specializing in emotional intelligence and SEL. The activities in the program include multiple teaching methods and techniques (such as role playing, creative drama, educational games, brainstorming, stations, talking circle, case study, discussion, and problem solving). The prepared activities were implemented in a sharing circle session order. The sharing circle is a powerful and versatile method in this practice (Schilling, 2001).

The researcher in this study acted as a participant observer, as defined by Hesse-Biber (2017). In this role, the researcher takes notes on the activities and engages in discussions with the participants during the program design and implementation. During the implementation of the emotional intelligence training program, the researcher fulfilled her role by taking notes of the environmental situations and participating in discussions. The observations made during this process referred to some points stated by Patton (2014). These points were the characteristics of the environment in which the program is implemented, the characteristics of the participants (such as in-group communications, gender-related behavior patterns, decision-making patterns), program activities, participant behaviors, planned and unplanned activities, body language and inconspicuous elements. The purpose of keeping these notes was to identify overlapping aspects between the findings and participant feedback, as well as to identify any missing or overlooked points. This would help to revise the next session before implementation and improve the reliability and validity of the analysis.

A total of 45 activities (9 of which are warm-up activities) developed by the researcher together with the resources examined and utilized from the literature on emotional intelligence skill areas were included. These activities were designed by taking into consideration the characteristics of the group to which the program would be implemented and by transforming them into a structure that was applicable at the primary school level and would be suitable for pre-service teachers to use in their classrooms in the future. Thus, the activities became both a tool for pre-service teachers’ own development and a material that they can apply at the primary school level by adding different dimensions.

To meet the course requirements and integrate the session content with material design, the pre-service teachers designed six main materials and several by-products for the activities. Additionally, they developed brochures and posters to introduce social–emotional skill areas as end-of-term materials. Table 4 lists the designs included in the implementation program along with their names and suitability for the session objectives.

**Table 4:** Materials designed for sessions.

Sessions	Designed materials
Recognizing and managing emotions Self-awareness and self-consciousness	Recognize Your Emotions (Game Design)
Communication skills	Communication Barriers (Poster Design)
Empathy skills	Newspapers Speak! (Cartoon Design)
Decision-making skills	Decisions and Results! (Poster, Slogan, Poem, Essay)
Problem/conflict-solving skills Stress coping skills	Rescue the Hanging Object! (Mechanism Design)
Group work and cooperation	Draw, Paint, Tell! (Story Design)

### 3.4 Data analysis

#### 3.4.1 Quantitative data analysis

The effectiveness of the intervention was measured using analysis and one-factor analysis of covariance (ANCOVA) analysis to ensure statistical validity. Before the selection and analysis, the participants who participated in both the pre-test and post-test were identified (23 = Control group, 22 = Experimental group). The obtained inventory data were entered into the SPSS software; incomplete data and scales with outliers were excluded (four scales each were excluded from both the groups). After the exclusion process, the data from the remaining 37 scales (18 = Experimental group, 19 = Control group) were analyzed using the Shapiro–Wilk test as the size of each individual group and the sum of both groups was less than 50 (18 < 50, 19 < 50, 37 < 50). The results of normality tests for the Bar-On Emotional Quotient Inventory data at the group level found *p*-values of 0.476 > 0.05 and 0.518 > 0.05 for pre-test data of the experimental and control group, respectively, and *p*-values of 0.935 > 0.05 and 0.769 > 0.05 for the post-test data, respectively; the scores seem to be normally distributed. ANCOVA analysis steps and results are presented in the “results related to quantitative data obtained from the scale used before and after the implementation” section.

#### 3.4.2 Qualitative data analysis

The two-stage coding strategy used with grounded theory was adopted, and content and document analysis and descriptive analysis were utilized. First, the focus group interviews were transcribed, reviewed, read, and confirmed by the participants. Similarly, participant diaries and process evaluation questionnaires were also read and categorized by associating them with each other using the “memoing” technique. This was followed by initial coding and focused coding. The same process was followed for participant diaries and focus group interviews. The results obtained were constantly compared with each other and interpreted and the results were explained with direct quotations. NVivo 12 application was used for data analysis. The transcripts of the focus group interviews were coded by two coders (one of whom was a researcher and the other a field expert) who were familiar with and had previous experience in the subject. Miles and Huberman’s formula was used for inter-coder consistency, and the percentage of inter-coder agreement was found to be 0.91.

## 4 Results

This section presents the findings obtained by analyzing the data collected separately and simultaneously within the scope of the embedded mixed design and integrating the results. In the last part, the analyses were synthesized as meta-inferences.

### 4.1 Results related to quantitative data obtained from the scale used before and after the implementation

Analyses found that the Skewness and Kurtosis (+/−1) values of the pre-test and post-test scores of the experimental and control groups were within the desired ranges (see Table 5). Then, a one-way analysis of variance (ANOVA) was conducted on independent samples to determine if there was a difference between the two groups in terms of pretest scores, which served as the control variable for the ANCOVA analysis. The results showed that the *p*-value (*p* = 0.648) was greater than 0.05, indicating that there was no significant difference between the two groups. A scatterplot was used to test the assumption stated for ANCOVA that ‘the control variable and the dependent variable should be linearly related in each group’ (Pallant and Manual, 2007). It was observed that there was a linear relationship between the control variable (pre-test score) and the dependent variable (post-test score). Additionally, Levene’s *F* test was conducted to examine the homogeneity of variances between the experimental and control groups in relation to the dependent variable. Based on the results of Levene’s test, the variances of the scores of the experimental and control groups in the post-test, which is the dependent variable, were found to be homogeneous [*F*_(1, 35)_ = 0.930, *p* > 0.05]. Finally, a two-way ANOVA test was used to assess the assumption of equality of the regression slopes between the confounding variable and the dependent variable in each group. The results showed that the interaction between the group variable and the pretest ‘group*pretest’ was not significant [*F*_(1, 33)_ = 1.615, *p* > 0.05]. Therefore, it can be concluded that the assumption of equality of the slopes of the regression lines was met. The ANCOVA tests and procedures were performed, and all analysis assumptions were met.

**Table 5:** Pre-test–post-test descriptive statistics and skewness-kurtosis values of emotional intelligence scores of experimental and control groups.

Pre-test							
Group		*N*	Median	Mean	*Sd*	Skewness	Kurtosis
Experimental	Emotional intelligence score	18	316.00	315.48	27.87	0.005	1.142
Control	Emotional intelligence score	19	319.00	319.55	25.90	0.129	−1.140

Post-test							
Group		*N*	Median	Mean	*Sd*	Skewness	Kurtosis
Experimental	Emotional intelligence score	18	334.37	334.02	24.53	−0.069	−0.911
Control	Emotional intelligence score	19	317.87	323.70	22.07	0.303	−0.424

**Figure 3:**
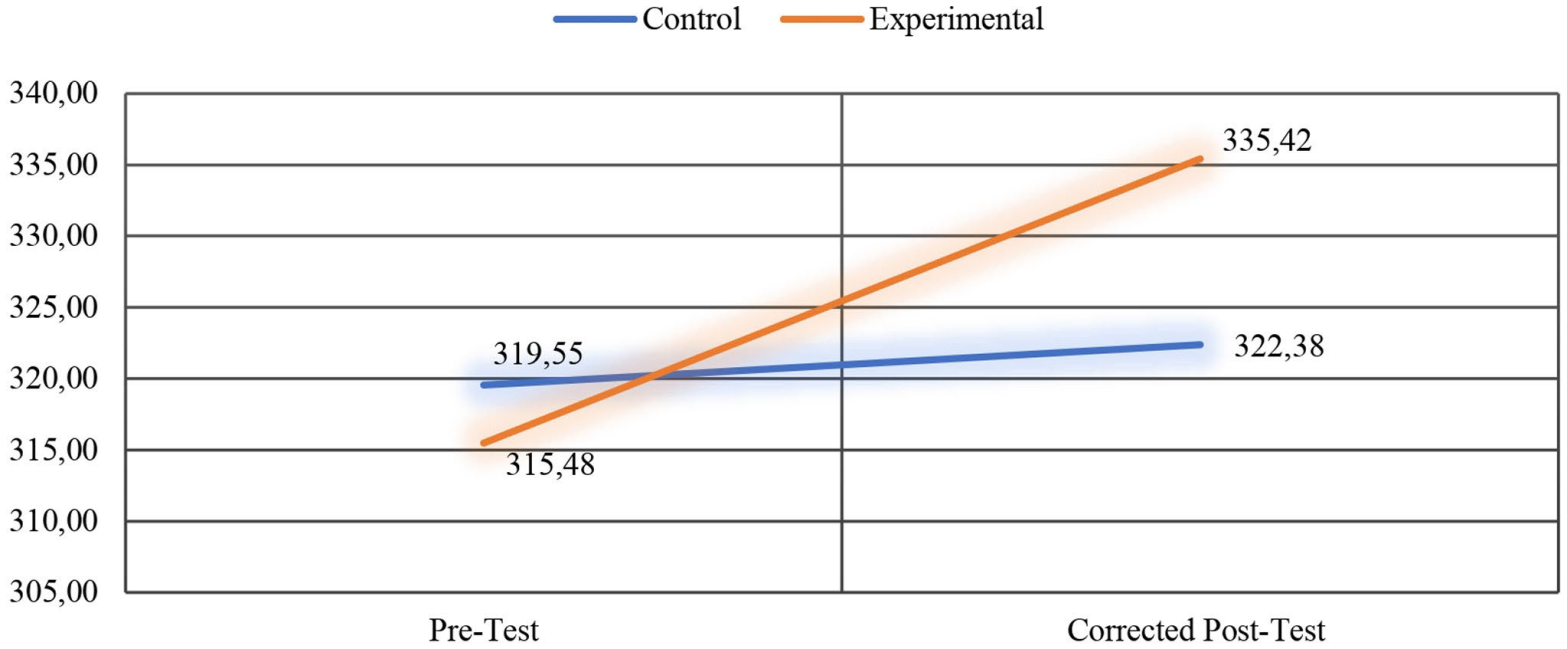
Line graph for the pre-test and corrected post-test scores of the experimental and control groups.

Researchers performed an ANCOVA to determine whether there was a significant difference between the post-test scores of the pre-service teachers in the experimental and control groups, which were adjusted according to the pre-test scores they received from the Bar-On emotional intelligence scale (Table 6). Additionally, Figure 3 presents the line graph showing the change in the pre-test and corrected post-test scores of the experimental and control groups.

**Table 6:** Pre-test, post-test, and corrected post-test scores of the experimental and control groups.

Group	*N*	Pre-test		Post-test		
		*X*	*Sd*	*X*	*Sd*	Adjusted mean
Experimental	18	315.48	27.87	334.02	24.53	335.42
Control	19	319.55	25.90	323.70	22.07	322.38

As Table 6 presents, the post-test scores of the experimental and control groups were 335.42 and 322.38, respectively, after checking the pre-test scores. Notably, there is a difference between the corrected post-test scores of the groups. To understand whether this difference is significant, it is necessary to look at the ANCOVA results. Table 7 presents the results of the ANCOVA together with the mean and standard deviation values of the groups, *F* value, *p*-value, and effect size eta squared (*η*^2^) value.

**Table 7:** ANCOVA results of the post-test scores of the experimental and control groups adjusted according to the pre-test.

Source	Sum of squares	*df*	Mean square	*F*	Sig.	*η^2^*
Corrected model	12287.111	2	6143.556	27.129	0.000	0.615
Intercept	3385.917	1	3385.917	14.952	0.000	0.305
**EI pre-test**	**11302.304**	**1**	**11302.304**	**49.909**	**0.000**	**0.595**
Group	1563.385	1	1563.385	6.904	0.013	0.169
Error	7699.568	34	226.458			
Total	4018275.744	37				
Corrected total	19986.679	36				

The bolded part shows the results of the analysis of the effectiveness of the experimental process. EI, emotional intelligence.

According to Table 7, when the pre-test scores were checked, it is noted that the training program for the development of emotional intelligence focused on SEL provided a significant increase in the emotional intelligence levels of the pre-service teachers in the experimental group [*F*_(1,36)_ = 49.909, *p* = 0.000 (*p* < 0.05), *η*^2^ = 0.595]. To determine the source of the difference between the groups, the Bonferroni test was performed, and it was determined that the difference was in favor of the experimental group. The analyses show that the training program applied to the pre-service teachers in the experimental group was more effective in the development of emotional intelligence than the current training program applied to the control group. Considering the effect size it can be said that it has a “large effect size” (Büyüköztürk, 2017).

### 4.2 Results related to qualitative data obtained during the implementation process

During the implementation process, the researchers analyzed the participant diaries and the results of the focus group interviews conducted at the end of each session. In some diaries, for some sessions, participants described only what was done for the process, while in others, they mentioned both what was done, how it contributed to the participant, and what awareness it raised. Only the parts of the diaries describing the process were not included in the analysis. During the analysis of the diaries, the statements written by the pre-service teachers about the activities within the sessions were grouped under two categories: “contributions” and “awareness.” Statements written for some sessions were only included in the category of contributions (problem/conflict-solving skills, coping with stress, and working and cooperating with groups). Statements with a frequency of 1 are not shown in the table. According to the data obtained from the participant diaries for the sessions, Table 8 presents the contributions and Table 9 presents awareness with frequency values. In addition to the participant diaries, the participant comments obtained from the focus group interviews conducted with pre-service teachers are presented together with the participant diary excerpts.

**Table 8:** Participant diary analysis results by session (contributions).

Sessions	Contributions	*f*
Self-awareness and self-consciousness	Seeing what values others attach importance to.	9
	Developing CV writing skills.	7
	Getting to know others better.	6
	Understanding the importance of values in life.	5
	Being able to see their shortcomings.	5
	Getting to know oneself better.	4
	Developing self-confidence.	2
Recognizing and managing emotions	Learning ways to cope with anger.	7
	Seeing the effects of anger on the individual.	5
	Seeing the significance of body language in communication.	4
	Seeing the similarity of individuals’ reasons for anger.	3
	The ability to cooperate.	2
	Learning unknown aspects of others.	2
	Developing imagination.	2
Communication skills	Learning communication barriers.	16
	Learning the types of communication.	2
	Being able to solve problems by talking.	2
Empathy skills	Creative thinking.	3
	Predicting one’s feelings and thoughts from their appearance.	3
Decision-making skills	Creative thinking.	4
	Seeing one’s own decision-making processes.	4
	Developing the ability to persuade.	3
	Thinking logically.	2
	Thinking fast.	2
Problem/conflict-solving skills	Learning conflict resolution strategies.	23
	Learning conflict types.	7
	Being able to put forward different ideas.	5
	Thinking fast.	3
	Ensuring cooperation.	3
	Being able to produce solutions to problems.	2
	Seeing which strategy is effective according to the types of conflict.	2
	Learning new things from each other.	2
	Making quick decisions together.	2
Stress coping skills	Relaxation by overcoming stress and excitement with breathing exercises.	8
	Learning ways to cope with stress.	4
	Realizing the significance of calm thinking.	2
Group work and cooperation	Ensuring cooperation.	16
	Developing the ability to work in a group.	16
	Creative thinking.	11
	Thinking fast.	9
	Developing in-group communication skills.	6
	The ability to make joint decisions.	3
	The ability to improvise.	3
	Improving attention and concentration.	3
	Developing self-confidence.	2
	Being able to use time efficiently.	2
	Active listening.	2
	The ability to dramatize.	2

**Table 9:** Participant diary analysis results by sessions (awareness).

Sessions	Awareness	*f*
Self-awareness and self-consciousness	Realizing that one does not even know their close friends.	11
	Realizing how much people know each other.	6
	Realizing the need to know oneself well.	3
	Recognizing the social–emotional skills possessed.	2
Recognizing and managing emotions	Realizing that in some situations body language is more important than words.	6
	Realizing that one should use gestures and facial expressions correctly.	3
	Realizing that most people cannot control their anger.	2
Communication skills	Realizing that communication barriers can be reduced through effective listening.	11
	Realizing that the more effective listening, the better the understanding.	8
	Recognizing the importance of finding a common language for strong communication.	7
	Recognizing the importance of careful listening in communication.	4
	Realizing that body language is more effective than verbal language.	3
	Realizing that in some situations body language is more important than verbal language.	2
	Recognizing the importance of using the right words in communication.	2
Empathy skills	Realizing that it is difficult to put oneself in their parents’ shoes.	11
	Recognizing the importance of empathy.	3
Decision-making skills	Recognizing how the brain (thoughts) affects perceptions.	8
	Realizing that people make their decisions under the influence of someone or a group (their environment).	7
	Realizing that you need to believe in order to succeed.	7
	Recognizing that group decisions are different.	4
	Realizing that one makes decisions more easily on their own.	4
	Realizing the need to make decisions by thinking ahead (future).	2

According to Tables 8, 9, based on the examination of the contributions and awareness of the self-awareness and self-consciousness session, the pre-service teachers especially made statements about seeing their deficiencies in terms of getting to know each other and realizing which values they have.

*An excerpt from the activity titled “Me according to you, you according to me (Who am I?)” from the PD3 coded diary: “Thanks to this event, we learned many characteristics, hobbies, and phobias of our friends that we thought we knew. Maybe through this event we will communicate better or look for ways to do so.” An example statement from the participant coded EP3 is, “…We realized that we did not know even the smallest details about the people we thought we knew. For example, one of them mentioned their hometown. Many of us wondered whether they were from there or not. In fact, we should have known that they were from that place. We realized that we did not know even the smallest detail…”*

Although the session on recognizing and managing emotions mostly contributed to learning ways to cope with anger, statements suggest that in some cases, body language was more important than words.

*An excerpt from the activity titled “What does my body language tell me?” from the PD17 coded diary: “Actually, what we say verbally is very ineffective compared to what the body language says. That is why what we do matters more than what we say. Therefore, we should pay attention to our gestures and facial expressions when communicating.” An example statement from the participant coded EP2 is, “…I learned how to calm our anger when we get angry, what to do when we get angry. The simplest thing is to take a deep breath. We had meditation music on, for example, and we imagined a space where we could close our eyes and relax. This way, we can calm our anger. We learned these things…”*

Statements indicate that the most important contribution of the communication skills session was learning about communication barriers and that it raised awareness that communication barriers can be reduced through effective listening.

*An excerpt from the activity “Listen and record!” from the PD27 coded diary: “Communicating effectively will give healthier results about people’s psychology. When we listen to the other person effectively, when we focus on the issue, we can get more logical results. When we exhibit this behavior, we gain the respect of both ourselves and the other person in terms of personality.” An example statement from the participant coded EP7 is, “…The most important thing I learned in this audio recording activity is that listening is the most important thing in communication. A big part of good communication is definitely listening well to the other person…”*

The empathy skills session contributed more to creative thinking and raised awareness about how difficult it is to put oneself in someone else’s shoes.

*An excerpt from the activity titled “Put yourself in your parents’ shoes” from the PD32 coded diary: “I think it was a good event. I think empathy is one of the skills that children need to acquire. If a child learns to empathize, he/she will approach people and events more moderately.” An example statement from the participant coded EP6 is, “…The activity of putting them in their parents’ shoes, I could not really visualize that moment. Really, it is a very heavy feeling, I mean really, what can you say, it is a very different feeling. You understand when you try to empathize…”*

It was stated that the decision-making skills session contributed to creative thinking, seeing their own decision-making processes, and developing persuasion skills. In terms of awareness, the following statements stand out: how the brain affects perceptions, decisions are mostly made under the influence of someone or a group (environment), and one needs to believe in order to succeed.

*An excerpt from the activity titled “Journey time!” from the PD1 coded diary: “In terms of decision-making, I sometimes make decisions on my own about an event, depending on the issue. Sometimes, I am influenced by a group or an individual. This event made me realize this; I was sometimes, not often, making decisions under the influence of an individual or under the influence of a group.” An example statement from the participant coded EP7 is, “…We learned that something actually influences our decision-making. We all have different needs and needs influence decision-making. We also learned that the environment influences our decisions. Because the guidance of our friends, what they said made us change our decision or make us more confident in our decision…”*

Statements indicate that the problem/conflict resolution skills session contributed most to learning conflict resolution strategies and types.

*An excerpt from the activity titled “Produce a solution!” from the PD12 coded diary: “The lesson I learned from this event is that even if there is a negative situation, even if we are in a difficult situation, we should use common sense in communicating with people. We should pay attention to the tone of voice and empathize with the other person from their point of view. As our ancestors say,* ‘*a soft answer turneth away wrath.’” An example statement from the participant coded EP5 is, “… We did not know much about conflict types and conflict resolution strategies; we did not know them at all. Thanks to you, at least we learned what the types of conflicts are. There were reenactments and things like that, and they helped us a lot. It was a very effective learning for us because before we did not know what they were, we learned about their types and so on…”*

Findings suggest that the stress coping skills session contributed to relaxation and learning ways to cope with stress by overcoming stress and excitement through breathing exercises.

*An excerpt from the activity titled “Journey to dreams with breathing exercise” from the PD2 coded diary: “In this event, I learned that we can actually feel better about ourselves by taking many things that we think are out of our control under our control. Stress was one of them. Once again, I realized that everything is in one’s hands and that one only has to want to do something.” An example statement from the participant coded EP7 is, “…These activities we did definitely did not only stay in the classroom. I think it is also reflected in our life outside. Whether it is coping with stress, whether it is breathing exercises. Because I really applied what I learned when I needed to…”*

It was repeatedly expressed that the group work and collaboration session contributed to collaboration, developing group work skills, and creative and quick thinking.

*An excerpt from the activity “Marshmallow Challenge” from the diary PD32: “I plan to use this activity when I become a teacher. This is because the materials needed for the activity are easily available and are of great interest to the students. For this reason, I think that the student’s interest in this activity will be intense and this game is a group game. In other words, they act with the consciousness of being a group based on cooperation. During this activity, one person’s idea can be linked to other ideas like a chain. I would also like to use this activity in my professional life as the students will enjoy doing something together.” An example statement from the participant coded EP3 is, “…I think the biggest secret to success in the Marshmallow activity is that the groupmates are very connected to one another and soothe each other’s stress at that moment. For example, in our group people were worried that other groups did the activity better. I said, ‘Guys, do not look at them, do what you are doing’…”*

The examination of the participant diaries of the pre-service teachers revealed that evaluations of the entire process were also included. These assessments found that the following words were mentioned in all 32 participant diaries in many different sections:

- It is fun,
- Efficient,
- Enjoyable,
- There is active participation, and
- It is educational.

Similarly, expressions in terms of the entire process frequently repeated the following words/phrases:

- Making information permanent with activities,
- To be able to produce creative products with the materials at hand,
- To be able to create a product as a group, and
- Learning the points to be considered when preparing materials.

Observations suggest that the data obtained from the participant diaries and the focus group interviews conducted throughout the process overlap and support each other.

### 4.3 Results related to qualitative data obtained after the implementation

The findings obtained from the focus group interview conducted with pre-service teachers (7 people selected for the focus group interview) to evaluate the process at the end of the implementation process were categorized under six categories: “circle practice” “sense of belonging” “peer pressure” “educator’s role” “feedbacks” and “materials” with reference to the semi-structured interview form questions. Table 10 presents the categories with their codes and these codes are explained with the excerpts obtained from the interviews.

**Table 10:** Prospective teachers’ views on evaluating the implementation process.

Categories	Codes
Circle practice	Determining the most effective type according to the scope of the activities through trials in inner and outer circle implementation.
	Providing a more intimate environment.
	Ensuring respect for ideas.
	Developing communication skills.
	Self-development.
	Being able to see each other’s faces.
	Being able to use body language comfortably.
	Maintain eye contact.
	Active participation.
	Interactivity.
Sense of belonging	Getting the right to speak.
	Finding opportunities to collaborate.
	In-group discussion.
	Gaining confidence by presenting ideas.
	Participating in activities as the whole class.
Peer pressure	Having different characters (e.g., dominant characters) can sometimes be a bit oppressive.
	While there is hesitation and pressure when presenting ideas, this feeling disappears as the process progresses.
	Preventing pressure by gaining self-confidence through getting to know each other and establishing cooperation.
	Developing a positive attitude through in-group activities and not feeling peer pressure.
Educator’s role in the process	Like a friend.
	They are one of the best people to communicate with.
	Easy to communicate with.
	They do not underestimate the ideas and products presented.
	Supportive.
	They have different ideas.
	Hard-working.
	Someone you feel comfortable expressing yourself.
	They help one gain self-confidence.
	They increase voluntary participation in practices.
	They present a smiling face.
	They provide a comfortable and free environment.
	Very good command of the classroom.
	They present a productive lesson process.
Educator feedback	A smile.
	Encouraging reinforcements such as “well done, successful, and congratulations”.
Designed materials	It is great when ideas become tangible objects.
	The instant design of materials in the classroom is constructive and effective in terms of creative and quick thinking.
	The materials made each week provide an active process.
	Being student-centered.
	Recognition of the value of the work done by creating products as a group.

The examination of Table 10 suggests that the pre-service teachers stated features such as providing a sincere environment, organizing according to the activities, respecting different ideas, and providing active participation.

*An example statement from the participant coded EP1: “…For example, I think that the communication between my classmates has increased. I had the opportunity to talk to a person I had never spoken to in class before when we did the circle activity in that lesson. Sometimes when we were divided into groups, I think I improved more in doing things as a group and I realized that I communicated more with my friends…”*

It is noticeable that pre-service teachers gained a sense of belonging through actions such as collaborative work, presenting their ideas comfortably, and in-group discussions.

*An example statement from the participant coded EP4: “…Since the number of the groups was not large, I mean, since there were four or five of them, everyone had the opportunity to speak. And everyone contributed here. For example, in the Marshmallow activity, we all held one end of the pasta. It was not like one person did not hold it, or one person held it and the others moved away. We all had to do it. For this reason, we all carried out a joint work…”*

While the pressure of different characters was felt during the process of working with the group in the activities, statements suggest that these negative situations were eliminated as they got to know each other and socialized during the process.

*An example statement from the participant coded EP3: “…In the activities we did at the beginning, of course, things happened. Because everyone’s personality is different. Some of them have too many leadership qualities. For example, they say, ‘let it be me, what I say is better.’ But we can actually overcome these in a way with the activities we do in the group. For example, we said ‘This is their personal characteristic, we have to give them their due, this is the leader, that is, he has more leadership qualities, they are right.’ We had such events. I think this is a normal situation…”*

Statements indicate that the educator has some characteristics such as being like a friend, having good communication, and being supportive and sensitive to different ideas in the process.

*An example statement from the participant coded EP4: “…Because you were friendly, we were able to talk about everything in the classroom environment without any problems. However, if you were a despotic person, if during those activities we said, ‘I should not say this, the teacher will be very angry if I say this, so I will keep quiet’ then I think most of the activities would have passed quietly. There would have been no participation. I thought there was no participation due to pressure. But because there was no pressure, because there was a comfortable free environment, everyone was able to speak, and everyone participated. I mean, I did not see anyone not attending the class. The whole class was active…”*

It was observed that the feedback received from the educator was mostly smiles and encouraging verbal reinforcements.

*An example statement from the participant coded EP1: “…The best thing is a smile, a well done. I think this is a very good reinforcement. These were good, motivating…”*

Finally, according to the opinions about the designed materials, statements suggest that creative and quick-thinking skills were developed by transforming different ideas into concrete objects and that the materials were student-centered.

*An example statement from the participant coded EP7: “…I think it was much more effective to do those materials in the classroom. Because we were making decisions very quickly as a group. We were developing new ideas and coming up with beautiful things. Maybe if we went and did it on our own, in the dormitory or at home, we would not have come up with such good ideas. But we were doing it in cooperation within the class. At that moment, really good ideas were coming out, because there was mutual interaction, there was communication. Everybody was using each other’s ideas and I think it was much more effective and beautiful…”*

The data obtained from the focus group interviews conducted with the pre-service teachers at the end of the implementation process coincided with and supported the data obtained from the participant diaries and the focus group interviews conducted throughout the process.

### 4.4 Results obtained by integrating qualitative and quantitative data obtained throughout the process with different methods

In this part of the findings, the aim is to see to what extent the quantitative and qualitative data support each other when integrated and to what extent the qualitative results confirm the quantitative results. For this purpose, the findings and results obtained from quantitative and qualitative dimensions were integrated and meta-inference was made. As a result of the quantitative data analyses conducted to test the effect of the social and emotional learning-oriented emotional intelligence development training program on increasing the emotional intelligence levels of the pre-service teachers in the experimental group and the qualitative data analyses conducted on the findings obtained from the documents collected for the evaluation of the effectiveness of the sessions and the focus group interviews, one can say that the program is an effective approach to increase the emotional intelligence levels of pre-service teachers.

## 5 Discussion

In line with the purpose of this study, the findings obtained from the quantitative part of the study showed that the implementation of the training program for the development of emotional intelligence focused on SEL was an effective approach in increasing the emotional intelligence levels of the pre-service teachers in the experimental group. Based on the Bar-On emotional intelligence model, studies have shown an increase in emotional intelligence competencies. These results confirm the results of other studies using similar models (Dolev and Leshem, 2016; Dolev and Leshem, 2017) and samples (Joshith, 2012; Gilar-Corbí et al., 2018). The ANCOVA determined that the implementation of the training program for the development of emotional intelligence focused on SEL was an effective approach in increasing the emotional intelligence levels of the pre-service teachers in the experimental group [*F*_(1,36)_ = 49.909, *p* = 0.000 (*p* < 0.05), *η*^2^ = 0.595]. The results obtained from the analysis of the participant diaries collected at the end of each session and the data obtained from the focus group interview during the implementation process showed that the findings obtained through quantitative methods were confirmed and supported by the statements regarding the positive developments and awareness in emotional intelligence skill areas. The findings obtained from the interviews conducted to evaluate the effectiveness of the implemented program demonstrated that the quantitative results were supported and explained. According to these results, the program was effective in ensuring the development of the emotional intelligence of pre-service teachers in the experimental group.

When the participant diaries obtained from the pre-service teachers for the evaluation of the sessions during and at the end of the implementation process of this study were integrated with the qualitative data obtained from the focus group interviews, the results were summarized as follows: Pre-service teachers better knowing and understanding both oneself and others, realizing the aspects that are lacking or need to be improved in a personal sense, turning negative emotions into positive ones and managing them, realizing the importance of body language and using it correctly, learning communication types and barriers, realizing how important it is to listen effectively, these include understanding the importance of empathy, realizing that needs and the environment are effective in decision-making, learning conflict types and conflict resolution strategies, creative thinking, quick thinking, group thinking, learning ways to cope with stress, cooperation, and gaining the ability to work in groups. At the same time, the study determined that emotional intelligence creates and develops awareness in all of the skill areas, teaches ways to make life more qualified with new learning, teaches how a teacher can effectively teach a lesson, and creates positive contributions and awareness such as teaching while entertaining, creative thinking, and active participation. The developed program was effective in ensuring the emotional intelligence development of pre-service teachers, the document and interview data obtained from the process were helpful in explaining this effect, and the integrated findings coincided with, confirmed, and supported each other.

Many studies support these findings. Academic and organizational efforts on emotional intelligence have shown that emotional intelligence can be developed in adults (Cherniss and Goleman, 2001; Goleman et al., 2002; Sala, 2002; Slaski and Cartwright, 2003; Bar-On, 2006; Boyatzis, 2009). Based on these findings and the relationship between emotional intelligence and effective teaching, it is suggested that teachers may also benefit from developing emotional intelligence (Haskett, 2003; Brackett et al., 2009). All those support the view that teacher-directed emotional intelligence development is an important element of SEL programs (Weare and Gray, 2003; Brackett et al., 2007, 2009; McCown et al., 2007; Palomera et al., 2008). In particular, teachers who develop their own EQ competencies can model desired EQ behaviors, apply EQ principles to everyday situations, and facilitate interpersonal problem solving and conflict resolution (Elias et al., 1997; Jennings and Greenberg, 2009).

The results obtained were compared with the results of studies conducted both with higher education students other than pre-service teachers and with teachers. For example, upon examining the results of the studies conducted with university students (from the fields of agriculture, dentistry, science and literature, education, engineering, nursing, social services, medicine, psychology, and business administration), it was determined that at the end of the training programs applied for the development of emotional intelligence, the trainings were effective in terms of developing emotional intelligence (Chang, 2006; Krishnamurthi and Ganesan, 2008; Fletcher et al., 2009; Karahan and Yalçın, 2009; Nelis et al., 2009; Jdaitawi, 2012; Erkayıran, 2016; Orak et al., 2016; Pertegal-Felices et al., 2017; Şen, 2017; Geßler et al., 2020). After similar implementation processes, a significant increase was observed in the emotional intelligence development of teachers (Tuyan, 2003; Hen and Sharabi-Nov, 2014; Dolev and Leshem, 2016, 2017; Turi et al., 2017; Sarısoy and Erişen, 2018; Martyniak and Pellitteri, 2020). One of the most cited studies on emotional intelligence training in higher education is Nelis et al. (2009), a study of an emotional intelligence training program for undergraduate students based on Mayer and Salovey’s (1997) emotional intelligence model. This program includes four 2.5-h sessions where students are given short lectures, role plays, group discussions, readings, and journal entries. This intervention showed improvements in performance-based measures of emotional intelligence even after 6 months compared to a control group. Later, Pool and Qualter (2012) developed an undergraduate course on the emotional intelligence model that includes short lectures, video clips, case studies, group tasks, role plays, an art gallery visit, and reflective journal entries. The results showed a positive change in understanding and managing emotions compared to a control group, again suggesting that emotional intelligence training courses in higher education can be successful. Further, Grant et al. (2014) trained undergraduate students in a single workshop on the Mayer and Salovey (1997) model of emotional intelligence, followed by a series of reflective writing journals over a three-month period, the results of which showed improvement in emotional intelligence (Joseph et al., 2019). It was observed that in addition to in-class applications, online applications also produced similar positive results (Gilar-Corbí et al., 2018; Durham et al., 2023). Although emotional intelligence development programs have been created for various groups and their effectiveness has been studied, there is limited research on their impact on pre-service teachers.

This study found that it is possible to provide emotional intelligence development with the program integrated into the curriculum without interfering with the academic performance of pre-service teachers and without burdening their out-of-class studies. These findings confirm and support the quantitative results. Similar research results also support that pre-service teachers’ emotional intelligence can be improved with additional intervention practices without affecting their curriculum (Joshith, 2012; Gilar-Corbí et al., 2018). However, it is noteworthy that emotional intelligence training applied as separate interventions or integrated into the course produces different results. In some studies (e.g., Potter, 2005), emotional intelligence training applied as a separate intervention does not fully include elements related to emotion. In some emotional intelligence training practices that are integrated into the course (e.g., Bond and Manser, 2009), little time is allocated to the integrated lesson itself, and extra tasks are given that burden the student. As a result, students’ academic achievement decreased and there was no improvement in emotional intelligence. Conversely, in some studies, it was possible to improve emotional intelligence without affecting students’ academic performance and without burdening their out-of-class studies (Gilar-Corbí et al., 2018). Similarly, the emotional intelligence training by McEnrue et al. (2009) designed for use in a leadership development program for business school students can be adapted for almost any context. Additionally, the workshops in the emotional intelligence training program that Grant et al. (2014) prepared for social work students can be easily adapted to other disciplines. Brackett and Katulak (2006) found that a comprehensive program focusing on developing emotional intelligence in K-12 settings improved the emotional intelligence of both students and educators. Thus, they showed that this program can be transformed to be used in higher education. A good example of integrating into different curricula is the work of Reilly (2005). Reilly provided an example of how a standard negotiation role used with law students can be adapted to emotional intelligence through reflection and discussion. He pointed out that in this way traditional classroom training exercises can be uniquely adapted for emotional intelligence training.

It is pleasing to see that the implementation principles and results of the reviewed studies show similar results to the implementation process and results of this study. One can conclude that development is inevitable if the designed programs are implemented with the right implementation steps. This way, there is a great expectation to encourage the development of such programs and to compare the results of their implementation. Although there are not many, the positive results of the implementation activities for pre-service teachers are pleasing. To ensure teachers’ effective professional development, it is necessary to integrate social–emotional skills training into the curriculum from the beginning of teacher education (Palomera et al., 2008; Pertegal-Felices et al., 2011). The social–emotional understanding thus formed can affect both relationship management and social awareness, helping teachers’ personal and professional development (Kaur and Sharma, 2022). Teachers who can recognize their social–emotional well-being can successfully implement SEL interventions in their classrooms (Hen and Goroshit, 2016).

## 6 Conclusion and recommendations

The results found the developed training program to be an effective approach to improve emotional intelligence among pre-service teachers. Moreover, data obtained from documentary sources and focus group interviews during and after the application of the program confirmed and adequately explained the quantitative results. The role of teachers in society’s reconstruction is significant. To meet the demands of a constantly evolving workforce, teachers responsible for educating young people must develop their leadership and mentoring skills. Therefore, emotional intelligence training should be included in teacher education. While there is limited evidence to support the inclusion of such training in school curricula, it is a necessary step toward preparing teachers for the challenges of the modern workforce. The study’s findings suggest a need to restructure the curricula for both classroom and subject teachers. The program’s integration and implementation in courses can also be guided by these findings. The study has yielded valuable qualitative findings on pre-service teachers’ acquisition of self-awareness, recognition and management of emotions, and communication, empathy, decision-making, problem-solving, conflict-solving, stress-coping, group work, and collaboration skills. This section explains the empirical results of the study and suggests adding emotional intelligence training programs to the curriculum or integrating them into courses. Evidence supporting the implementation of emotional intelligence activities, developed in accordance with course objectives using various teaching methods and techniques, can also be found in previous studies (Reilly, 2005; McEnrue et al., 2009). On the other hand, the study’s qualitative findings reveal the thoughts and actions of pre-service teachers in the classroom. These results provide valuable evidence on how and to what extent the neglected emotional factor may have a role in the effectiveness of education. The study’s findings suggest that methods, techniques, and activities prioritizing emotional intelligence components should be incorporated into educational planning.

As a general result, this study found that it is possible to provide emotional intelligence development with the program integrated into the curriculum without interfering with the academic performance of pre-service teachers and without burdening their out-of-class studies. This study is limited to the literature within the scope of the subject examined for the developed program and the eight skill areas addressed within the scope of emotional intelligence. It is recommended to develop new programs for emotional intelligence development based on different emotional intelligence models. This study was designed for pre-service teachers studying in the classroom teaching program. The researchers believe that it would be useful to design it for different teaching fields. Activity-based course contents containing emotional intelligence components can be created for the curricula of all branches of the faculty of education, especially classroom teaching. One limitation of the study is the sample size. Working with larger samples may increase the generalizability of the results. Another limitation of the study is that it is not known whether the success of the program will continue in the long term. Therefore, a longitudinal version of the study would be useful in terms of its results. This study examined the overall level of emotional intelligence. Analyzing emotional intelligence sub-dimensions may yield specific results. Additionally, comparing teachers who receive emotional intelligence training during their education with those who do not may be insightful. It would be interesting to analyze the classroom activities of these teachers in relation to emotional intelligence components. It may be recommended that the designed program be adapted and applied to other levels, especially primary school. Due to its structure, the program can be used in educational environments as well as in course environments that aim to provide personal development. Teachers can request practices can be requested as in-service training. Evidence-based practice and evaluation should be continuous. It is recommended that Ministries of Education and funders invest more in the creation and implementation of new SEL programs to raise the level of achievement.

## Author’s note

This article was produced from Meryem Özdemir Cihan doctoral dissertation completed in 2020 (Thesis No: 657056).

## Data availability statement

The raw data supporting the conclusions of this article will be made available by the authors, without undue reservation.

## Ethics statement

The studies involving humans were approved by Atatürk University Rectorate, Social and Human Sciences Ethics Committee, and Educational Sciences Unit Ethics Committee. The studies were conducted in accordance with the local legislation and institutional requirements. The participants provided their written informed consent to participate in this study.

## Author contributions

MÖ: Conceptualization, Data curation, Formal analysis, Investigation, Methodology, Resources, Supervision, Validation, Visualization, Writing – original draft, Writing – review & editing. MD: Conceptualization, Investigation, Validation, Writing – review & editing.
